# Caatinga Revisited: Ecology and Conservation of an Important Seasonal Dry Forest

**DOI:** 10.1100/2012/205182

**Published:** 2012-08-01

**Authors:** Ulysses Paulino de Albuquerque, Elcida de Lima Araújo, Ana Carla Asfora El-Deir, André Luiz Alves de Lima, Antonio Souto, Bruna Martins Bezerra, Elba Maria Nogueira Ferraz, Eliza Maria Xavier Freire, Everardo Valadares de Sá Barreto Sampaio, Flor Maria Guedes Las-Casas, Geraldo Jorge Barbosa de Moura, Glauco Alves Pereira, Joabe Gomes de Melo, Marcelo Alves Ramos, Maria Jesus Nogueira Rodal, Nicola Schiel, Rachel Maria de Lyra-Neves, Rômulo Romeu Nóbrega Alves, Severino Mendes de Azevedo-Júnior, Wallace Rodrigues Telino Júnior, William Severi

**Affiliations:** ^1^Departamento de Biologia, Universidade Federal Rural de Pernambuco, Rua Dom Manoel de Medeiros, s/n, Dois Irmãos, 52171-900 Recife, PE, Brazil; ^2^Unidade Acadêmica de Serra Talhada (UAST), Universidade Federal Rural de Pernambuco (UFRPE), Fazenda Saco, s/n, 56.900-000 Serra Talhada, PE, Brazil; ^3^Departamento de Zoologia, Centro de Ciências Biológicas Universidade Federal de Pernambuco (UFPE), Avenida Professor Moraes Rego, 1235-Cidade Universitária, 50670-901 Recife, PE, Brazil; ^4^Direção de Ensino/Gerência de Pesquisa e Pós-Graduação-Cidade Universitária, Instituto Federal de Pernambuco-Reitoria, Campus Recife, Avenida Professor Luis Freire 500, 50740-540 Recife, PE, Brazil; ^5^Laboratório de Herpetologia, Departamento de Botânica, Ecologia e Zoologia, Centro de Biociências, Universidade Federal do Rio Grande do Norte, Lagoa Nova, 59072-900 Natal, RN, Brazil; ^6^Departamento de Energia Nuclear, Centro de Tecnologia, Universidade Federal de Pernambuco (UFPE), Avenida Professor Luís Freire 1000, Cidade Universitária, 50740-540 Recife, PE, Brazil; ^7^Programa de Pós-Graduação em Ecologia e Recursos Naturais, Centro de Ciências Biológicas e da Saúde, Departamento de Ecologia e Biologia Evolutiva, Universidade Federal de São Carlos, Rodovia Washington Luiz Km 235, 13565-905 São Carlos, SP, Brazil; ^8^Unidade Acadêmica de Garanhuns, Universidade Federal Rural de Pernambuco, Avenida Bom Pastor, s/n, Boa Vista, Heliópolis, 55296-901 Garanhuns, PE, Brazil; ^9^Departamento de Biologia, Universidade Estadual da Paraíba, Avenida das Baraúnas 351, Bodocongó, 58109-753 Campina Grande, PB, Brazil; ^10^Departamento de Pesca e Aquicultura, Universidade Federal Rural de Pernambuco, Rua Dom Manoel de Medeiros, s/n, Dois Irmãos, 52171-900 Recife, PE, Brazil

## Abstract

Besides its extreme climate conditions, the Caatinga (a type of tropical seasonal forest) hosts an impressive faunal and floristic biodiversity. In the last 50 years there has been a considerable increase in the number of studies in the area. Here we aimed to present a review of these studies, focusing on four main fields: vertebrate ecology, plant ecology, human ecology, and ethnobiology. Furthermore, we identify directions for future research. We hope that the present paper will help defining actions and strategies for the conservation of the biological diversity of the Caatinga.

## 1. Introduction

What is the current status of biodiversity research in the Brazilian Caatinga? In an attempt to resolve this issue, a team of researchers recently used the biological group “insects” as a proxy to estimate the status of biodiversity research in this environment [1]. Although this paper is very relevant and interesting, it attempts to extrapolate the condition of biodiversity research in a large region from the information available for insects alone.

In Brazil, the word Caatinga is used to designate a large geographic area comprising a variety of different types of vegetation. It is also used to name the semiarid region that occupies the largest portion of the northeast of Brazil. Its temperature and rainfall qualify the area as a tropical seasonal forest. Most of the information collected in studies of the Caatinga vegetation only applies to a small number of areas. It is difficult to formulate generalizations on the vegetation dynamics of this region because of the lack of replication, which is common for other vegetation types throughout the world. The study of the vertebrates inhabiting the Caatinga follows the same trend found in the study of the vegetation. Overall, sample size and sampling effort are often not satisfactory. For this reason, many researchers worldwide have adhered to the idea of maintaining an information network, such as TROPI-DRY, to aid in the preservation of Neotropical dry forests, such as the Caatinga. This particular region includes very large disturbed areas as well as areas undergoing desertification processes, both of which have been given high priority for preservation and/or restoration [2]. Information networks such as TROPI-DRY combine ecological research, remote sensing, and social sciences through the use of standardized protocols that allow for comparisons between different tropical dry forest areas.

Despite the recognized gaps in our knowledge of this region [3–5], the number of studies on Caatinga biodiversity has increased considerably over the past decades. In this paper we have made a comprehensive analyzes of a number of important studies carried out in the last 50 years about flora, vertebrate fauna, human ecology, and ethnobiology in the Caatinga. We aimed to summarize information on these subjects as well as identify key directions for future research. To compile data on the aforementioned topics, we used seven main databases (Biological Abstracts, Google Scholar, SciELO, Scirus, Scopus, JSTOR, and Web of Science) as well as several book chapters published on these topics. We hope that the present paper will help defining actions and strategies for the conservation of the biological diversity of the Caatinga.

## 2. Vertebrate Ecology

### 2.1. Ichthyofauna

Ichthyofauna are found in all aquatic environments across a wide range of habitats. It is the group that possesses the greatest richness among vertebrates. In this regard, there are 2,587 species of fish that are endemic to freshwater environments in Brazil [6].

Rosa et al. [7] have suggested that the fish fauna in the Caatinga have not yet been well documented; however, the authors highlight that various studies have been conducted in a few locations and this information is still restricted to monographs, dissertations, and environmental diagnostics for the construction of dams, which cannot be found online. In addition, several of these studies are rather specific, which does not allow for a general view of the diversity of fish fauna in this environment.

In recent years, several studies have increased our knowledge of the diversity of fish species in the Caatinga by providing descriptive studies, such as those concerning the annual species of the Rivulidae family, including Costa [8, 9], Costa and Brasil [10], and Costa et al. [11, 12]. Rosa et al. [7] regarded the Caatinga as the site with the greatest biodiversity of fish from the Rivulidae family in relation to other ecosystems. Rivulids, or annual fish, are known by their distinct life cycle; the adults develop rapidly and deposit their eggs during floods to avoid desiccation during the dry season [9].

Rosa et al. [7] and Rosa [13] reported the occurrence of 240 fish species in the Caatinga distributed across seven orders, with Siluriformes and Characiformes as the most diverse ones (101 and 89 species, resp.). Compiling the most recently published studies, this richness increases with the addition of four new species in the northeastern portion of the São Francisco River basin: *Aspidoras psammatides* [14], *Pimelodus pohli *[15], *Simpsonichthys harminicus* [16], and *Salminus franciscanus* [17]. Some additional species from the Siluriformes order have been recorded, such as *Platydoras brachylecis *in the Parnaíba River [18] and *Rhamdiopsis krugi *[19] in the caves of Chapada Diamantina National Park, demonstrating the preferences of some fish species for more sheltered habitats.

Medeiros and Maltchik [20] and Luz et al. [21, 22] noted that temporary environments such as lakes and tributaries are important marginal environments for shelter, feeding and rest for several fish species. There is an urgent need for preservation of the integrity of these habitats and their functionality, owing to their role in conservation of fish diversity in the Caatinga.

Based on this panorama of findings, the following challenges have been detected in relation to our knowledge of the ichthyofauna of the Caatinga: (i) long-term research to determine the actual diversity of fish species in this ecosystem; (ii) a taxonomic review of the systematically ambiguous groups; (iii) the characterization of the natural history of the fish species of the Caatinga, especially those that are involved in local economy; (iv) more precise monitoring of the consequences of introductions of exotic species, which requires the design of more efficient conservation projects to protect these species and the ecosystems within which they live.

### 2.2. Herpetofauna

The herpetofauna includes Anurans, Gymnophiona, Testudines, Squamata, and Alligators. These groups display a significant amount of variability in their morphotypes, that is, associated with specific ecological niches [23]. These organisms are also highly susceptible and sensitive to environmental changes [24, 25]. The first continuous efforts to monitor the herpetofauna of the Caatinga were initiated in the 1970s and 1980s [26–31] and were especially concerned with the herpetofauna in the states of Pernambuco and Paraíba.

Since then, the number of studies on the herpetofauna of the Caatinga has increased. However, the group is still underrepresented because it encompasses a large diversity of unknown morphotypes [32–34], and those studies that have been performed have concentrated on only a few locations [32, 33, 35–38]. Research from the past three decades has revealed increases in the number of occurrences of new herpetofaunal species [36, 37, 39–44] as well as in the occurrences of described species [26, 45–62]. This work has helped to fill in geographical gaps but confirms the need for further studies to elucidate the full diversity of the group [32].

Despite its underrepresentation, the known herpetofauna of the Caatinga currently comprises 175 species (53 anurans, 3 Gymnophiona, 7 testudines, 47 lizards, 10 Amphisbaenia, 52 serpents, and 3 alligators). Approximately 12% of these species are endemic [33–38].

It is worth stressing that public policies providing financial support for multidisciplinary projects involving other taxa have also contributed to the increase in the number of publications concerning various aspects of the herpetofauna of the Caatinga, including studies providing specific species inventories (*n* ≈ 35), studies on the ecology (*n* ≈ 22) or conservation (*n* ≈ 22) of herpetofauna, descriptions of new species (*n* ≈ 20), reports of the geographic distribution of certain populations (*n* ≈ 12), and, much less frequently, the behavioral biology and thermoregulation of herpetofaunal species (*n* ≈ 5).

Lizards and serpents are the subjects of more than 50% of the scientific articles on the herpetofauna. Members of the Testudines order and crocodilians have been poorly studied, with occasional records of these species occurring in the main drainage basins of the Caatinga [36, 63–66]. Ecological research on the Gymnophiona order (amphibians) and the Amphisbaenia (Squamata), exclusively fossorial animals, is nonexistent, and there are few studies on frogs in the larval (tadpole) stage [35]. The large gap in knowledge concerning these animals is perhaps the result of the very low number of specialists in Brazil and/or the methodological difficulties involved in performing systematic research [36]. There is a clear need for financial incentives and training of herpetological specialists. The Caatinga herpetofauna is poorly understood compared to those found in other parts of Northeast Brazil, such as the Atlantic Forest [67, 68].

### 2.3. Avifauna

The Caatinga has been identified as an important center of endemism for South American birds [69–72]. Although the avifauna is the most well-known animal group with respect to its taxonomy, geographic distribution, and natural history, there are still many gaps in our understanding of the distribution, evolution, and ecology of the avifauna of the Caatinga as compared with other natural regions of Brazil, such as the Atlantic Forest and the Cerrado [73–76]. In fact, these latter regions have been disproportionately investigated because the majority of the universities in Brazil are concentrated in the southeast and south regions of the country; a greater number of researchers are concentrated in the southeast region, and the majority of theses and dissertations are conducted in central south Brazil [77].

Most manuscripts that have been published on the avifauna of the Caatinga are inventories containing annotated lists of important species (endemic and threatened species); few studies have addressed issues at the community ecology level or analyzed the biology of specific bird species [74, 76, 78–83]. Published studies that investigated the evolutionary, behavioral, or reproductive biology of avifaunal species or examined their conservation potential are rare. In addition, few studies have analyzed the seasonal movements of species or the interactions among the avifauna of this region and the availability of resources, which encompass topics such as pollination, dispersion, and foraging ecology [84–89].

For decades, the avifauna of northeast Brazil was described only in the writings of foreign naturalists and preserved as material deposited in scientific collections. The majority of this material, however, was derived from the avifauna of the Atlantic Forest [90]. In the past 50 years, Brazilian ornithology has made great advances with respect to our knowledge of the birds of the Caatinga; however, the most significant advancements occurred in the past two decades. Altogether, 60 ornithological studies were conducted in the Caatinga between 1961 and 2011. The first study, published exactly 50 years ago, was the result of four expeditions performed in the states of Ceará, Alagoas, Paraíba, Bahia, and Piauí during 1957 and 1958 [91]. These expeditions surveyed portions of the Caatinga and the Atlantic Forest.

In the 1970s, three studies were performed in the Caatinga. The first was conducted by Coelho [92], who recorded 273 bird species in northeast Brazil, mainly in the state of Pernambuco. The next two studies were performed in the states of Paraíba [93] and Bahia [94]. In the 1980s and 1990s, 13 additional studies were performed, covering the states of Pernambuco, Ceará, Paraíba, and Bahia [95–100]. Among these studies, the study by Coelho [101], which was conducted in the Biological Reserve of the Serra Negra (in a semiarid region of Pernambuco), is notable because it examined bird species of both the Atlantic Forest (damp vegetation) and the Caatinga (dry vegetation). The noteworthy studies by Azevedo Júnior et al. [102] and Azevedo Júnior and Antas [103, 104] also contributed to our understanding of the biology of the game species *Zenaida auriculata*, which suffers a great deal of pressure from hunting in the semiarid areas of the northeast region.

Since the early 2000s, with the increases in ornithologists, research incentives, and requirements that environmental licensing organizations monitor avifauna, new areas of the Caatinga have been studied in the states of Pernambuco (Vale do Catimbau National Park and other parts of the Legal Reserve), Ceará (Chapada do Araripe and the Aiuaba Ecological Station), Bahia (Raso da Catarina), and Rio Grande do Norte (Seridó Ecological Station), resulting in more than 34 scientific studies [105–109]. These studies, above all, record new occurrences of bird species, expanding the known distributions of several species across the Caatinga [110–116].

The studies published on the avifauna of the Caatinga facilitated the publication of three lists of avifaunal species between 1995 and 2003. In the first two lists, 338 and 347 species were reported, respectively [117, 118]. However, the bird species recorded in other vegetation enclaves that occur in isolation in the Caatinga region, such as *brejos de altitude* (highland forests), alpine pastures, and areas of ecological tension, were not included in the first two lists. Therefore, Silva et al. [75] compiled a third, more exhaustive, list for the region; this list contains 510 bird species, distributed across 62 families, and includes the avian species of the aforementioned enclaves so as to improve estimates of regional species density and because of their influence on ecological processes such as dispersion, which helps to maintain the distribution of the avifauna across the Caatinga. Since the last of these three lists was published, only *Petrochelidon pyrrhonota *has been added to the list of birds of the Caatinga [114].

Although the diversity of the Caatinga avifauna may be relatively well established, there remains a need for further scientific research to determine the influence of seasonal variations in resource availability on the dynamics of bird populations and communities, both terrestrial and aquatic, particularly with regard to the mechanisms that contribute to the spatial-temporal heterogeneity that exists across the Caatinga. Filling in these knowledge gaps is extremely important for conservation initiatives and the management of Caatinga bird populations, which suffer from increasing anthropogenic pressures and are safeguarded in only a small number of protected areas [4, 120].

### 2.4. Mammals

Over the last four decades, notable progress has been made in expanding our knowledge of mammals of the Caatinga. The notable research efforts of the 1980s contributed information on the species richness, ecology, behavior, physiology, and distribution of mammalian fauna in the Caatinga [119, 121–124]. From this series of studies, the following five initial assumptions concerning the biology and ecology of the mammals of the Caatinga were made: (i) the number of species is relatively low, (ii) there is a low degree of endemism, (iii) the mammals of the Caatinga represent a subset of the mammals of the Cerrado, (iv) the mammals living in the Caatinga do not possess pronounced physiological adaptations to life in this semiarid region, and (v) these mammals exhibit behavioral adaptations that allow them to live in this environment.

Although extremely important, some of the results obtained by these authors have been amended by recent studies. For instance, the total number of species described by Willig and Mares in 1989 [119] increased from 80 to 101 in a study published by Fonseca and colleagues in 1996 [125]. More recently, Oliveira [126] added 47 new species to the list. This number increases even more if we consider the studies performed by Gregorin and Ditchfield [127], Gregorin et al. [128], Feijo et al. [129], and Moratelli et al. [130]. Together, these studies added six new species of bats to the Caatinga. Furthermore, Canale et al. [131] and Ferreira et al. [132] recorded the presence of two new primates inhabiting the Caatinga. Collectively, these studies added nine new species that were not listed in the Oliveira [126] study, thus bringing the current total number of mammalian species living in the Caatinga to 156.

In 1989, the rodent *Kerodon rupestris* was the only mammalian species known to be endemic to the Caatinga [119]. However, in recent years, an additional 12 species have been defined as endemic, including ten rodents (*Wiedomys pyrrhorhinos*, *Trinomys yonenagae*, *Trinomys albispinus minor*, *Trinomys albispinus sertonius*, *Thylamys karimii*, *Dasyprocta* sp. n., *Oryzomys* sp. n., *Oxymycterus* sp. n., *Rhipidomys* sp. n. ssp. 1, and *Rhipidomys* sp. n. ssp. 2; Oliveira [126]); one primate (*Callicebus barbarabrownae*; Oliveira [126]); two bats (*Xeronycteris vieira *and *Chiroderma* sp. n; Gregorin and Ditchfield [127], and Gregorin et al. [128], resp.). As a result of these more recent findings, it is now accepted that there is a considerable richness of rodent species in the Caatinga [133]. As for bats, although studies remain scarce [134], it appears likely that the richness of bat species in this ecosystem will increase with further research.

The number of known mammalian species in the Caatinga (*n* = 156) is greater than that of the Pantanal (*n* = 113; Meserve [135]) or the Grande Chaco (*n* = 102; Meserve [135]), but it is less than that of the Amazon Forest (*n* = 350; Meserve [135]), the Atlantic Forest (*n* = 261; Ribeiro et al. [136]), and the Cerrado (*n* = 199; Klink and Machado [137]). There is a notably high level of endemism in the Amazon Forest (*n* = 205, 58.6%; Meserve [135]) relative to the Cerrado (*n* = 18, 9.3%; Marinho-Filho et al. [138]) and the Caatinga (*n* = 12, 7.7%). All of these recent results show that the Caatinga has a species richness that, though not comparable to the Amazon or Atlantic Forests, is higher than that found in other ecosystems and clearly higher than previously thought. The new data also highlight the inadequacy of the initial assumption regarding the mammals of the Caatinga as a subset of the mammalian species found in the Cerrado (e.g., Oliveira et al. [139]).

Life in semiarid conditions imposes constraints and limitations on many mammalian species. However, by means of water deprivation experiments, Streilein [123] discovered that there were no pronounced physiological adaptations in several small mammalian species living in the Caatinga. More recent studies support the earlier findings (e.g., Mendes et al. [140]; Ribeiro et al. [141]). According to Streilein [123], small mammals exhibit behavioral responses to compensate for limiting factors in the semiarid environment. As an example, Streilein [123] mentions the preference of some species for mesic and rocky areas (see, however, Freitas et al. [142]). Recent studies have confirmed the importance of behavior in the adaptation of a number of mammals to the Caatinga. For example, the endemic species *Trinomys yonenagae *digs holes in dunes to escape the hottest period of the day [143]. In addition to the thermoregulatory benefit of this behavior, Santos and Lacey [144] suggest that it also serves as protection against predators. Furthermore, Moura [145] proposed that *Sapajus libidinosus *(previously known as *Cebus libidinosus*; *C. apella* in Oliveira [139]) uses its high cognitive capacity to overcome the difficulties associated with obtaining food in the Caatinga.

Despite the advances in our knowledge of the mammals of the Caatinga since the first important assumptions were made decades ago, many geographical areas of the Caatinga have not yet been studied. This deficiency indicates a high potential for a further increases in knowledge concerning the richness and geographical distributions of mammalian species, not to mention their ecology and behavior. It has been recommended that researchers avoid examining locations close to cities and villages to prevent any methodological bias associated with these areas (Oliveira [139]). Moreover, Bernard et al. [134] made other recommendations that, although proposed for bat fauna studies, may be extrapolated to studies of other mammals; these suggestions include long-term research incentives involving previously studied areas; increasing the number and frequency of studies on the mammalian species present in collections and museums; investing in the training of taxonomists; and creating an online database to record and organize the occurrences and distributions of mammalian species in the Caatinga. Naturally, these efforts will only be viable if there is an attendant commitment to efficiently protect the regions established as priority areas for the conservation of the mammals of the Caatinga [126].

## 3. Plant Ecology

To date, at least 248 studies have examined the Caatinga vegetation, 33% of which attempted to answer questions related to the flora and the phytosociology of the region. This focus reflects the nation's policies that support increases in scientific and technological knowledge. Before the 2000s, these policies predominantly encouraged research aimed at identifying the composition of woody species and characterizing the structure of the communities in the areas considered to be protected or subject to a very low amount of anthropogenic intervention [146–152].

Despite the differences that exist among the sampling criteria and the sampling efforts of the various floristic and phytosociological studies of the Caatinga [146, 147], these studies confirm that the vegetation displays various types of physiognomies, ranging from predominantly herbaceous vegetation to arboreal vegetation, with differences in floristic composition among the physiognomic types, and includes a considerable number of endemic species [147, 149, 152]. However, there is still a large gap in our understanding of the richness of both the herbaceous component and the climbing and epiphytic plants [147, 148, 153], which are estimated to number approximately 1500 species, or threefold as many than the known richness of woody species [154].

Beginning in the 2000s, there was a large increase in the number of studies aimed at understanding the ecological and ecophysiological processes that influence the establishment and renewal of plant populations. Among these studies, those related to pollination, the reproduction system, and plant phenology make up the greatest percentage (17%) [155–161].

In general, the plant species in tropical dry forests exhibit diverse phenological patterns [162, 163] that reflect the heterogeneity in the environmental factors that create gradients in resource availability, such as precipitation, temperature, photoperiod, and soil type [162]. These forests generally encompass a range of deciduousness, from fully deciduous species to evergreen species [164]. However, despite the rich phenological diversity of tropical dry forests, little is actually known about the driving factors and mechanisms by which plants adapt their phenological patterns. This situation is even more complicated in the Caatinga, a dry tropical forest that occupies one of the largest land areas in the world and exists at the extreme of water resource availability for forests [165]. The necessity of collecting phenological information for these areas is reinforced by the high degree of anthropogenic disturbance to which these forests have been subjected [166] and the potential risks of probable climate changes and further reductions in water resource availability [165].

The timing of phenological events can be critical to the reproduction, establishment, and survival of plants [167]. Therefore, an understanding of the mechanisms and factors that influence these events and their relationships to pollinating and dispersing agents may help to inform better biodiversity management and conservation planning efforts. According to Quesada et al. [168], tropical dry forests are highly dependent on pollinators (54–80% of the species are self-incompatible), and in the case of the Caatinga vegetation, Machado and Lopes [169] found that 98% (*n* = 147) of the plant species they examined were pollinated by animals, with entomophily as the predominant means of pollination.

Of the phenological studies published about the Caatinga, five examined groups of 10 to 20 woody species [156, 158, 170–172] and two other studies investigated one and a few woody species, respectively [173, 174]. Only one study examined two herbaceous species [175]. Several other studies dealt exclusively with aspects of reproduction [155, 157, 169, 176–180]. In total, these studies considered only a small proportion (<10%) of the Caatinga flora, which contains more than 1500 species, even when only considering the most typical vegetation types [154].

The few phenological studies undertaken in the Caatinga region have indicated that precipitation is the major driving factor of phenophases [181]. Precipitation controls the phenology of numerous species, although other species initiate their phenophases independent of the occurrence of rainfall [157]. Amorim et al. [156] demonstrated that different species show different responses to soil water availability, with some budding in response to sporadic rainfall during the dry season, while others remain dormant. Recently, Lima and Rodal [158] reported a close relationship between plant phenology and wood density (the quantity of water stored in tree trunks). These authors noted that deciduous plants with low wood densities are capable of storing more water in their trunks and that budding and reproduction often occur during the dry season in species with low wood densities. In contrast, the deciduous trees with high-density wood tend to initiate their phenophases more in accordance with soil water availability. Lima [182] likewise determined that species with low-density wood maintained high water potentials throughout the year, although they only budded or flowered when the photoperiod increased (not necessarily when soil water was available). There have been few studies published in this area, and the effects of the photoperiod on Caatinga plants still need to be examined along latitudinal gradients and in terms of the synchrony of phenological events, which has been investigated in other dry tropical forests [183–187].

Future phenological studies in the Caatinga should not only focus on the relationships between the timing of phenological occurrences and rainfall, which have been examined numerous times [171, 181], but also investigate the biotic mechanisms responsible for the occurrence of various phenophases and attempt to integrate the diverse characteristics of a given plant species into a whole entity [188], with the aim of predicting possible phenological patterns from assemblages of such characteristics [168, 189, 190]. These types of studies would allow Caatinga species to be classified in terms of their functional phenological types, that is, as groups of species that demonstrate certain sets of characteristics and functions and respond in similar manners to multiple environmental factors [191], independent of their phylogenetic or taxonomic relationships [191, 192]. One paper [193] analyzed a large number of leaf, stem, and life-form characteristics to distinguish functional groups of species in the Caatinga, but phenological traits were not incorporated into the model.

Studies of phenological types can greatly contribute to our understanding of ecosystem functioning and biodiversity maintenance and should include (i) long-term studies [194]; (ii) environmental and latitudinal gradients and multiple successional stages [168]; (iii) experimental manipulations that simulate climatic variations, especially those related to soil water availability and longer dry periods; (iv) relationships between the physiology and phenology of plants [195]; (v) phenology and storage mechanisms; (vi) fruiting season and dispersion syndromes. Finally, (vii) the different Caatinga physiognomies should be characterized in terms of functional groups based on phenological and other plant traits.

In addition to the studies on the phenology and reproduction of plants in the Caatinga, other significant processes that influence the establishment of plants and the renewal of plant populations in the Caatinga have been investigated. Studies on seed germination constitute the greatest percentage (10%) of such studies [196–199], followed by studies of plant physiology and ecophysiology (8%) [200–203] and studies examining the dispersion and dynamics of the seed bank (6%) [204–207]. These studies demonstrate the following: (i) many species have seeds with a rigid tegument and seed coat dormancy, which help the plant embryo to avoid germination in the dry season and protect the seed from predation by small animals; (ii) leaf fall is the main survival strategy during the dry period, but leaf replacement can take place in the dry season given the occurrence of thunderstorms with intense rains; (iii) many adult plants of the Caatinga contain chlorophyll covered by a fine layer of rhytidome in their stems, leading to the hypothesis that in some woody (noncactus) species, photosynthesis is performed in the stem organ during the dry season, explaining the positive growth rate of the plants during the dry season; (iv) the dispersion of seeds is influenced by the seasonality of the region, with a predominance of zoochory recorded in the moister Caatinga areas; and (v) the seed bank dynamics suggest the existence of opposite tendencies among Caatinga areas, with greater seedling emergence occurring in either the dry season or the rainy season, depending on the area.

All of the aforementioned processes influence the dynamics of plant populations. However, few studies have been designed to model the dynamics that exist in the Caatinga. To date, only 12% of studies have provided information on the birth and mortality rates of the plant populations of the Caatinga; these studies mainly focused on the influence of regional climatic seasonality on herbaceous species [175, 196, 208–210]. No studies have been performed on the population dynamics of epiphytic or climbing plants.

It is important to stress that approximately 80% of the annual precipitation in the Caatinga is distributed irregularly and unpredictably within the rainy season [147, 152, 211], and any of the following scenarios may occur: (i) a prolongation of the duration of the rainy season or the dry season; (ii) erratic rainfall events during the dry season; (iii) droughts (dry spells) during the rainy season; though very rarely, (iv) years that lack a dry season entirely, particularly in the hypoxerophilous areas of the Caatinga nearest to the coast [200]. These rain distribution models act as a selective force because they influence the reproductive behavior of the plants and cause differential mortality in the plant populations, thereby constituting key factors that influence the dynamics of the ecosystem.

Plant birth is predictable over time both for herbaceous and woody species, with the predominant period of birth occurring during the rainy season or after the erratic rains occurring in the dry season [196, 212]. The latter incidents have a negative effect on the population because the seedlings die when the dry season resumes [175, 210]. Some populations display synchronous and concentrated births at the beginning of the rainy season, which may be disadvantageous if dry spells occur right after the beginning of the rainy period. Other populations distribute births among the months of the rainy season, a model that minimizes the mortality caused by the occurrence of water deficits [148, 196]. Thus, various birth distribution models may have evolved as strategies to escape the unpredictability of the rain distribution from year to year, indicating that any forecasts of the size of a given population, especially those species with a long-life cycle, must be based on data from a long-time series that incorporates the effect of this unpredictability.

Plant mortality in the Caatinga is unpredictable over time. Mortality may occur in any of the climatic seasons, and mortality rates vary among years [208, 210]. Nevertheless, marked differences in the timing of mortality have been observed between woody and herbaceous populations in the Caatinga. Herbaceous populations display heightened mortality in the dry season because of the predominance of the therophyte life-form [212]. The opposite has been recorded for woody plants because of the heightened recruitment of seedlings, a seasonal state that is delimited by the duration of the rainy period [148, 213].

The direct and indirect impacts of the rainfall distribution appear to function as the main causes of population mortality. At the transition between climatic seasons, when individual adults still display deciduous characteristics or have only undergone incomplete leaf replacement [156, 171], the force of the water can directly uproot young individuals in the soil [148]. Rainfall events can also indirectly cause mortality because, due to the weight of the rain, dry branches of woody plants may fall onto fragile seedlings, uprooting them from the soil. Vines, which grow rapidly after the arrival of the rainy season, may also uproot fragile plants by using the seedlings as support [211]. Another cause of mortality recorded during the rainy season is herbivory of newly recruited seedlings by wild fauna (populations of beetles, ants, and other organisms that grow in size during the rainy season).

Little importance has been placed on the influence of microhabitats on the vegetation of the Caatinga, but recent studies indicate that the microhabitats formed by cracks in rocks, at the edges of streams, or along stretches shaded by nondeciduous woody tree canopies can influence the dynamics of the vegetation of semiarid environments. These microhabitats serve as refuges, minimizing the severity of the dry season and facilitating the survival of individuals from various plant populations [175, 211]. These microhabitats may also influence plant growth rates, but this potential impact needs to be directly assessed in the Caatinga.

Few studies have characterized the processes of litterfall, nutrient cycling, biological fixation, and the environmental services provided by the vegetation of the Caatinga. Those studies that have been performed indicate that climatic seasonality is a highly significant factor that influences these processes and that not all legume species fix nitrogen [214–217].

Similarly, few studies have analyzed the impacts of different forms of management on the dynamics of the Caatinga plant populations or on the resiliency of disturbed areas. According to Castelleti et al. [218], management has increased in the Caatinga, despite the role of the plant populations in the local and regional conservation of the climate [219]. These studies have shown that plant management is mainly aimed at agropastoral activities and that abandoned areas exhibit a capacity for natural recovery, although the composition of the native species may be altered as a result of biological invasion, which can alter the abundances of various plant populations [220–224]. The land use history and the duration of use influence the recovery speed of habitats modified by human activity [225], but the parameters that can be used to indicate that a disturbed area has completed its recovery through natural regeneration processes have not yet been defined.

## 4. Human Ecology and Ethnobiology

By virtue of the adverse environmental conditions, a large portion of the human population living in semiarid regions develops strong relationships with the local floristic and faunistic resources [226]. Animals and plants of the Caatinga provide sources of food but also serve many other needs, such as medicinal remedies (medicinal plants and animals), leather, hide, and ornamental pieces (horns, hooves, eggs, and furs) as well as providing pleasure and decoration (e.g., canaries and other pets) [226–232]. Additionally, some animal species are persecuted and killed because of their conflicting relationships with the human population [226, 230, 233]. In this context, hunting in the Caatinga region has long been practiced and represents a traditional form of wildlife management.

Because of the cultural richness of the local population and their diverse interactions with the local fauna, the Caatinga is an extremely suitable area to conduct ethnozoological studies. These studies are of fundamental importance from a socioenvironmental perspective because factors such as excessive exploitation, hunting, and illegal trading of wild animals have been designated as threats to some vertebrate species. Nonetheless, in the past few decades, researchers have begun to systematically investigate the relationships between the local inhabitants and the wild fauna of the Caatinga region. Our review indicates that 92 studies on the ethnozoology of the area have been published to date (89% of these in the last ten years). The majority of these studies have examined the popular uses of medicinal animals (*n* = 25) and ethnoentomology (*n* = 22). The other topics examined include hunting (*n* = 9), ethnoornithology (*n* = 6), ethnoherpetology (*n* = 5), ethnoichthyology (*n* = 4), the ethnozoology of noninsect arthropods (*n* = 4), religious uses of fauna (*n* = 4), ethnocarcinology (*n* = 3), and ethnomastozoology (*n* = 3). Eight other studies covered topics that are not restricted to one zoological group. The majority of the investigations were performed in two states: Bahia (*n* = 52) and Paraiba (*n* = 21). It should be noted that, even within these two states, this area of research has been restricted to only a few areas. For example, in Bahia, more than 90% of the studies were conducted in two municipalities, Feira de Santana (*n* = 12) and Santa Terezinha (*n* = 20). These patterns provide evidence that the scarce ethnozoological research on the Caatinga has been restricted to a small number of themes and geographic areas.

Despite the scarcity of information, some patterns can be identified in the forms of the interactions between the local populations and various animal taxa. Among the invertebrates, bees stand out as an important group because of their honey production and medicinal uses. Vertebrates are the main hunting targets in the region. Mammals comprise the preferred sources of food because of their size and the possibility of a greater yield for the energy invested in hunting. The species hunted most commonly are *Dasypus novemcinctus* (nine-banded armadillo), *Euphractus sexcinctus* (six-banded armadillo), *Tamandua tetradactyla* (southern tamandua), and *Conepatus semistriatus* (striped hog-nosed skunk) [226, 231, 234, 235]. The populations of some animals that were previously common in the Caatinga, such as the deer *Mazama guazoubira* and *M. americana* (important game species in the region), appear to have declined in many areas [226, 228]. Despite the general preference for mammals as game, when considering the diversity of species used for food, the avifauna is actually the most notable group, particularly species of the families Columbidae and Tinamidae. These results appear to reflect the richness of these groups in the Caatinga, where 511 species of birds and 156 species of mammals have been recorded (see comments above).

Despite their value as a source of protein, the high frequency of game birds targeted is primarily related to their use as pets [229, 232, 236, 237]. This value represents a strong stimulatory factor for the illegal trade of birds in the Caatinga. Various cities in the interior of northeast Brazil have public markets and open fairs where birds and other wild animals are sold [237].

With regard to reptiles, few species are used as food. The lizard *Tupinambis merianae* has been identified as the species most frequently consumed. Although the practice is not common, other reptiles, such as *B. constrictor, Iguana iguana, *and* Caudisona durissa*, can also be eaten [230, 238]. Nonetheless, the principal practical value of reptiles appears to be related to the medicinal value of the products derived from these animals, and various species of chelonians, snakes, and lizards have been used as remedies by local populations [77, 227, 239–243].

Conflicts represent another important type of interaction between humans and animals in semiarid northeast Brazil [230]. The reasons for the conflicts, which lead to the killing of wild animals, include attacks on livestock, risk to human lives, destruction of crops, and risk of disease transmission. The principal taxa involved in conflicts with local inhabitants are reptiles (particularly snakes), mammals (particularly carnivores), and, to a lesser extent, birds (granivorous or falconiformes). Of these animals, reptiles tend to be the group most frequently considered dangerous and persecuted. Snakes, even the nonpoisonous species, are often beaten or killed when encountered. Medium- and large-sized carnivores, such as *Leopardus tigrinus*, *Puma yagouaroundi*, *Puma concolor*, and *Cerdocyon thous*, are also killed because they prey on domestic animals [226].

All forms of interaction between the fauna and inhabitants of the Caatinga require further investigation, particularly considering their importance for the development of management and conservation plans. Limited ethnozoological information is available for important groups of vertebrates, likely because of the legal implications surrounding the principal groups of game animals, which are protected by law. This situation influences the choice of topics for ethnozoological studies, most of which involve groups such as insects and fishes. In addition to the scarcity of ethnozoological studies concerned with important animal groups, there are gaps in knowledge from the geographical perspective because, even in the states for which a large number of studies have been performed, the studies have been restricted to two or three cities and then to the same community within these cities.

Most of the studies on plants in Brazil have focused on ethnobiology. For example, we found 156 articles on ethnobotany in Brazil published between 1992 and 2011. Of these, 75% were published between the years 2006 and 2011. When classified according to the ecosystem, a very large proportion of these studies focused on the Atlantic Forest (42.95%), followed by the Caatinga (30.13%), the Cerrado (16.67%), the Amazon (12.18%), the Pampas (4.49%), and the Pantanal (1.28%). This scenario, in which more studies have been performed in the Caatinga than the Cerrado or Amazon, is surprising in a way. Along with the Atlantic Forest, we expected these ecosystems to have hosted the greatest number of studies. In the case of the Cerrado, it is a biodiversity “hotspot” (as is the Atlantic Forest) and an environmental priority for conservation, given that it is the largest tropical forest on the planet, covering nearly half of Brazil.

Considering only the studies focused on the Caatinga, the scenario is similar to the national trend; that is, the majority of the studies (89.36%) were published during the same time interval mentioned above. This fact was also recorded by Oliveira et al. [244], who found a greater volume of ethnobotanical studies published in recent years, possibly due to the growing number of researchers (senior and junior) who have started working on these topics over the years. Furthermore, the Caatinga covers nine Brazilian states (eight in the northeast region and one in the southeast region), but the ethnobotanical studies have mainly concentrated on the state of Pernambuco, in which more than 65% of the studies on this region were conducted.

The majority of the ethnobotanical studies performed in the Caatinga are either directly or indirectly concerned with medicinal plants. Studies that express the intention of contributing to a foundation by searching for new biologically active molecules particularly stand out [245–248], as do studies focused on conservation and sustainable management [245–248] and the cognitive effects of medicinal plants [249, 250]. An example of a study that aimed to contribute to the search for new biomolecules is that of Araújo et al. [245], who applied syndromic importance as a tool in the search for plants in the Caatinga with high levels of tannins, optimizing the search for plant resources with high yields of this molecule. This method was made possible by the use of tools and/or concepts derived from other branches of knowledge, such as phytochemistry, chemical ecology, and pharmacology. Although medicinal plants have been considered with the greatest frequency, other ethnobotanical topics have been gaining ground, such as the research on wood forest products that emphasizes their uses and the impacts of harvesting on the native vegetation [251, 252].

In general, the ethnobotanical research conducted in the region has helped not only to clarify the biodiversity of useful plants of the Caatinga [246, 253] but also to determine the possible impacts that the collection practices may exert on the environment [251, 254]. One of the studies that compiled the medicinal plants used for traditional and nontraditional purposes in the Caatinga revealed that more than 350 taxa (including native and exotic plants) compose the popular pharmacopoeia of the local human populations [253]. With regard to the conservation of plant resources, ethnobotany has contributed by measuring the usage and availability of the plant species in the environment or rather by seeking to improve our understanding of how much the native local vegetation may provide without harming the wild populations [254–258]. In this regard, ethnobotanical research has made many contributions to this field. An example is the study of Oliveira et al. [254], who suggested priority species for management and conservation programs based on ecological data along with information on plant usage.

Another point worth stressing is that the majority of the ethnobotanical studies performed in the Caatinga have been performed in communities that are located close to the native vegetation areas. These populations clearly collect and use species from the neighboring vegetated areas for various purposes, such as medicine, wood (e.g., housing construction or to delineate territories), food, and fuel (firewood and charcoal). These studies have contributed to both an understanding of the local demands for the resources necessary for survival and the identification of the species/populations that suffer from the greatest anthropogenic pressure. These studies can further contribute to the formulation of proposals for the rational use and sustainable management of these species, thus contributing to the conservation of the local biodiversity.

Another notable research topic, though not relevant to the majority of the existing studies, is the testing of hypotheses and predictive models. In this regard, researchers from the region have tested various hypotheses/models, such as those associated with the appearance, diversification, and functional redundancy of species, in an attempt to explain patterns in the selection and usage of plants by humans. An example of a hypothesis that was conceived of and tested in an area of the Caatinga is the hypothesis of diversification, which asserts that exotic plants are inserted into a given community to supply therapeutic treatments for diseases for which the native plants are not effective. This hypothesis was corroborated in a study conducted in the Caatinga by the observation of significant differences in the presence of particular classes of secondary metabolites between native and exotic plants [259].

## 5. Perspectives

The heterogeneity in our understanding of the Caatinga is possibly what led Santos et al. [1] to assert that “objectively, society must rapidly reduce the institutional anemia experienced by some SDTFs [seasonally dry tropical forests] and other seasonal ecosystems by expanding local institutional capacity and research networks (i.e., aggressive capacity-building) with the task of (1) informing stakeholders [of] the costs and benefits from general land use patterns and those imposed by public policies, and (2) developing and transferring the better-practices required for using natural resources sustainably.”

Clearly, more studies need to examine the communities and populations of plants and animals of the Caatinga because generalizations on the dynamics of this type of vegetation must consider the differentiation of existing habitats, which in turn influences the ecophysiological behavior, reproductive dynamics, and survival capacity of the individuals of different populations. Much of this research has been financed by public organizations, but no policy exists to establish a central storage bank of field data. Such a database would enable subsequent analyses considering longer time series.

With regard to the flora of the Caatinga, many questions and gaps have been acknowledged in previous studies [4, 5, 213] but remain unanswered. For example, what is the annual contribution of seeds to the renewal of the seed bank in the Caatinga? Within a single population of Caatinga plants, is there temporal variation in sexual characteristics? What is the biological significance of the variation in the phenological and reproductive behaviors of plants for the population dynamics and evolution of the plants in this environment? What Caatinga species display interactive dynamic models, and how might these interactions facilitate our understanding of the ecosystem functions of the Caatinga? What survival mechanisms are employed by plants in the Caatinga? What species can be used to assist with the recovery of anthropogenically disturbed areas?

With regard to the fauna, the main challenges to be overcome in the next decades are as follows: (i) directing efforts for multidisciplinary studies with long-term research goals; (ii) promoting taxonomic reviews of species with many morphotypes; (iii) recording the real geographic distribution, diet, spatial-temporal specifics, and reproductive aspects of the species that occur in the Caatinga.

With regard to the conservation status of Caatinga species, there are a number of relevant issues, such as the consequences of climatic modifications and desertification processes for population structures, particularly given that while Caatinga species may be adapted to long periods of drought, certain species may be extremely vulnerable to rapid climate changes.

The gap in our knowledge concerning the resiliency of this ecosystem is substantial. It will be necessary to invest in studies examining the dynamics of the recovery of anthropogenically disturbed areas with regard to both the land use history and duration of use. Currently, the similarities and differences in the natural regeneration process between preserved and disturbed areas are poorly understood. Thus, it is difficult to contemplate and discuss strategies to recover disturbed areas, which are continuously increasing in area in this type of ecosystem because of the social demands and the technological development of the country.

In view of the current panorama of research findings, it is evident that future ethnobiological studies should be extended to a greater diversity of taxa. Such data would make it possible to confirm the ethnozoological and ethnobotanical patterns recorded to date and to search for answers to questions not yet resolved, including the following: are socioeconomic parameters (e.g., schooling, income, and age) the main factors influencing the interactions between animals and the local population? Or is hunting motivated by leisure and culture? Are cultural aspects more important than either of these factors? Does subsistence hunting persist only in more isolated sites? Are hunting activities more intense in well-preserved areas of the Caatinga? Do the types of uses of the fauna vary according to taxonomic group? Are hunting and the use of wild animals similar in urban and rural areas? Which forms of interactions/uses have the most negative effects on the species exploited? Is there a relationship between the uses of particular species and the availability of the local fauna? Does the illegal trade of wild animals persist because of cultural or socioeconomic aspects? We believe that these are some of the questions that should guide new ethnobiological studies. These studies should be performed with a greater taxonomic rigor, an aspect that is lacking in a large number of ethnozoological studies carried out to date.

Despite all of these knowledge gaps, the Ministry of the Environment has gathered together researchers to consider and propose priorities for the conservation of the biological diversity of the Caatinga [3, 219]. If, however, there remains too great of a gap in the scientific knowledge of the Caatinga [1], the proposed public policies may not achieve the stated goals and may need to be rethought.
